# Comprehensive assessment of executive functioning following childhood severe traumatic brain injury: clinical utility of the child executive functions battery

**DOI:** 10.3389/fpsyg.2023.1160210

**Published:** 2023-10-30

**Authors:** Mathilde Chevignard, Amanda Guerra, Rafika Fliss, Lucie Salah, Emmanuelle Pineau, Pauline Notteghem, Jeanne Roche, Caroline Huon, Agata Krasny Pacini, Didier Le Gall, Nathalie Fournet, Jean-Luc Roulin, Arnaud Roy

**Affiliations:** ^1^Rehabilitation Department for Children with Acquired Neurological Injury, Saint Maurice Hospitals, Saint Maurice, France; ^2^Sorbonne Université, Laboratoire d’Imagerie Biomédicale, LIB, CNRS, INSERM, Paris, France; ^3^Sorbonne Université, GRC 24 Handicap Moteur et Cognitif et Réadaptation (HaMCRe), Paris, France; ^4^Santos Dumont Institute (ISD), Macaíba, Brasil; ^5^Univ Angers, Nantes Université, LPPL, SFR CONFLUENCES, Angers, France; ^6^Les Capucins: Réadaptation Spécialisée Adultes et Enfants, Soins de Longue Durée et EHPAD, Angers, France; ^7^SMAEC Resource Center for Children, Adolescents, Young Adults With Acquired Neurological Injury, Miribel, France; ^8^Les Capucins: Réadaptation Spécialisée Adultes et Enfants, Soins de Longue Durée et EHPAD, Angers, France; ^9^Institut Universitaire de Réadaptation Clemenceau, Strasbourg, France; ^10^Centre Hospitalier Universitaire d'Angers, Angers, France; ^11^Laboratoire de Psychologie et Neurocognition, Université Grenoble-Alpes, Université Savoie-Mont-Blanc, CNRS, Grenoble, France; ^12^Centre référent des troubles d’apprentissage, Hôpital femme-enfant-adolescent, CHU de Nantes, France

**Keywords:** brain injuries, child, executive functions, performance-based tests, everyday life, adolescent, assessment

## Abstract

**Objectives:**

To perform a detailed description of executive functioning following moderate-to-severe childhood traumatic brain injury (TBI), and to study demographic and severity factors influencing outcome.

**Methods:**

A convenience sample of children/adolescents aged 7–16 years, referred to a rehabilitation department after a TBI (*n* = 43), was compared to normative data using a newly developed neuropsychological test battery (Child Executive Functions Battery—CEF-B) and the BRIEF.

**Results:**

Performance in the TBI group was significantly impaired in most of the CEF-B subtests, with moderate to large effect sizes. Regarding everyday life, patients were significantly impaired in most BRIEF clinical scales, either in parent or in teacher reports. Univariate correlations in the TBI group did not yield significant correlations between the CEF-B and socio-economic status, TBI severity, age at injury, or time since injury.

**Conclusion:**

Executive functioning is severely altered following moderate-to-severe childhood TBI and is best assessed using a combination of developmentally appropriate neuropsychological tests and behavioral ratings to provide a comprehensive understanding of children’s executive functions.

## Introduction

Traumatic brain injury (TBI) is the leading cause of pediatric death and lifelong acquired disability, representing a serious public health issue (Beauchamp et al., 2011; Lumba-Brown et al., 2018). Recent reviews estimated that the annual median incidence of pediatric TBI corresponds to 691 injuries per 100,000 per year (Ryan et al., 2015; Thurman, 2016), including 9/100,000 deaths. Among those who sustain a TBI in any given year, 74 out of 100,000 require hospitalization. Altogether, at least 80% of TBIs are mild, 13–17% are estimated as moderate, while severe TBI represents 3–7% of all TBIs in developed countries (Dewan et al., 2016). Severe childhood TBI often causes diffuse brain lesions including lesions of frontal regions and cortical-sub-cortical pathways that may interrupt the developmental trajectory of cognitive, emotional, behavioral, and psychological functions (Lindsey et al., 2019). Thus, the large majority of children who sustain severe TBI suffer persistent secondary disability (The Lancet, 2018), including sensory-motor deficits, cognitive, behavioral, emotional and adaptive impairments (Chevignard et al., 2020; Neumane et al., 2020).

Among cognitive functions affected by severe TBI, the executive functions (EFs) are often significantly affected (Ganesalingam et al., 2011; Lipszyc et al., 2014). These functions are defined as a collection of related but distinct cognitive abilities that allow individuals to engage efficiently and effectively in intentional, complex, purposeful goal-directed problem-solving actions, through conscious and effortful processing. They allow one to adapt to novel situations, especially when action routines and over-learned sequences are not sufficient (Anderson et al., 2004; Diamond, 2013).

Most studies recognize that EFs are considered a multidimensional rather than a unidimensional construct (Baggetta and Alexander, 2016). Among the different functions that are considered as EFs in different theoretical models, inhibition, working memory (or updating) and flexibility (or shifting) are regarded as the most basic and central EFs (e.g., Miyake et al., 2000; Lehto et al., 2003; Diamond, 2013; Friedman and Miyake, 2017). These basic components are implied in the operation of higher-level EFs, such as planning, reasoning, and problem solving (Diamond, 2013). Despite EFs components being considered as independent constructs, they are strongly interrelated (Miyake et al., 2000; Lehto et al., 2003; Diamond, 2013).

Executive functions develop throughout infancy, childhood, and adolescence, following different developmental trajectories, in parallel with maturation of prefrontal regions and cortical–subcortical pathways (Diamond, 2013). These immature areas of the brain are known to be especially vulnerable to the effects of early brain insult (Levin et al., 1991; Anderson, 2002). Further, the development of EFs is inextricably associated with the emergence of the other cognitive functions, such as language, visual–spatial skills, attention, processing speed, and memory, thus making it difficult to assess them and interpret test results when “lower-level” functions are impaired (Ewing-Cobbs et al., 2004; Levin and Hanten, 2005; Cermak et al., 2021). Executive dysfunction is among the most frequently reported area of neuropsychological impairment following childhood TBI, both in their cognitive and behavioral regulation components (Araujo et al., 2017; Chevignard et al., 2017; Keenan et al., 2017). EFs deficits have been shown to have significant and long-standing consequences on everyday functioning (e.g., independence for homework, transportation, meal preparation), social interactions, academic and social-professional achievement (e.g., ability to study, to obtain, and to maintain a job in adulthood; Gerrard-Morris et al., 2010; Kurowski et al., 2011; Petranovich et al., 2020).

Most studies report EF deficits in TBI of various severity levels, especially moderate and severe TBI (Babikian and Asarnow, 2009; Beauchamp et al., 2011; French et al., 2014; Krasny-Pacini et al., 2017; Le Fur et al., 2020). Overall, many children who suffer moderate-to-severe TBI present long-term impairments in processing speed, attention (Levin et al., 1991), working memory, and other EFs (Babikian and Asarnow, 2009). These deficits are not always evident initially, especially in the youngest children (Kurowski et al., 2011; Petranovich et al., 2020; Jones et al., 2021). They often emerge or become significant several months or years post-injury, when their expected maturation did not take place as expected, and environmental demands increase (Anderson et al., 2004; Babikian and Asarnow, 2009; Keenan et al., 2017).

Following pediatric moderate-to-severe TBI, few predictors have been identified that can reliably identify individuals at risk for long-term cognitive difficulties (Levin et al., 1991; Lumba-Brown et al., 2018). Children injured at a younger age, with lower pre-injury functioning and living in families from lower socio-economic backgrounds are more susceptible to worse EFs deficits (Levin et al., 1991; Anderson et al., 2001; Jones et al., 2021). Family factors, such as parenting style, stress burden, and parental/home stress, have also received additional attention and have been related to behavioral aspects of EFs (Levin et al., 1991; Schorr et al., 2020). There is also evidence for an effect of injury severity on EFs impairments, with some evidence for a dose–response relationship (Levin et al., 1991; Araujo et al., 2017; Le Fur et al., 2020).

Despite the recognition of executive deficits in children with moderate–severe TBI, the differentiation between specific executive component deficits and overall EFs deficits remains unclear regarding performance-based tests. In fact, the theoretical and methodological approach to assess EFs in children with TBI is often limited. Few studies simultaneously assessed at least the three basic EFs through performance-based tests (inhibition, working memory, and flexibility—; Treble-Barna et al., 2016; King et al., 2020), and the inclusion of more complex EFs (e.g., planning, problem solving) is scarce (Wade et al., 2010; Chavez-Arana et al., 2018). In this sense, understanding global or specific executive deficits on moderate–severe TBI in children remains a question to be addressed. In addition, studies rarely include different measures of the same EFs component - but with different characteristics (e.g., verbal/nonverbal; motor/cognitive approach)—to control for the effect of more basic processes on executive performance (Denckla, 1996; Roy, 2015).

Given the low ecological validity of available standardized tests for EFs (Gioia et al., 2010; Chevignard et al., 2012), a number of studies have also used questionnaire based reports of executive functioning in everyday life [e.g., the Behavior Rating Inventory of Executive Function (BRIEF) questionnaire; Gioia et al., 2000], either in addition to standardized testing, or sometimes exclusively (Chevignard et al., 2010, 2017; Donders et al., 2010; Gioia et al., 2010; Ganesalingam et al. 2011; Potter et al., 2011; Wilson et al., 2011; Krasny-Pacini et al., 2017; Le Fur et al., 2020; Maloney et al., 2020; Narad et al., 2020; Smith-Paine et al., 2020; Tramontana et al., 2021). However, given the low correlation between the BRIEF questionnaire and performance-based measures of EFs (Toplak et al., 2013), it remains crucial to comprehensively assess executive deficits using developmentally adequate tasks, in combination with questionnaires. In fact, experimental tests can be too unnatural and only assess executive functioning over a short period of time and questionnaires sometimes can be considered too subjective. To our knowledge, few studies reported multifactorial assessment of different EF components using both performance-based tests and rating scales (Treble-Barna et al., 2016; King et al., 2020). In addition, in these studies only one test per EF component was considered, limiting the control of the effect of non-executive components on executive tests. In this sense, studies providing comprehensive assessment of EFs, using developmentally adequate tests with robust psychometric and clinical validity are still scarce (Roy, 2015; Roy et al., 2020).

Therefore, the primary aim of this study was to perform a preliminary comprehensive assessment of executive functioning following moderate-to-severe childhood TBI, using a newly developed child neuropsychological performance-based test battery (Child Executive Functions Battery—CEF-B; Roy et al., 2020), designed to assess the four main executive domains in children and adolescents (inhibition, working memory, flexibility, and planning), using both verbal and non-verbal subtests. Additionally, we aimed to compare the results of the CEF-B to parental and teacher ratings of the BRIEF, and to explore the demographic and medical/TBI severity variables influencing CEF-B performance [i.e., family socio-economic status (SES), age at injury, time since injury, and TBI severity].

We postulated the following hypothesis: (1) all components of EFs would be impaired (in both CEF-B measures and BRIEF scores); (2) performance-based tests and rating measures would be partially congruent to discriminate executive deficits in children diagnosed with TBI; (3) younger age at injury, longer time since injury, greater injury severity, and lower parental SES would be associated with more severe EF deficits.

## Methods

### Participants

A convenience sample of children with moderate-to-severe TBI was included, according to the following inclusion criteria: children and adolescents (1) who sustained TBI before the age of 17 years, and who were subsequently referred to a pediatric rehabilitation department for assessment and/or follow up; (2) aged 7–16 years at assessment; (3) parents able to understand and read French; and (4) parents signed informed consent. Exclusion criteria were the presence of (1) a history of diagnosed developmental, neurological, or psychiatric disorders, (2) uncorrected sensory disorder (visual or hearing) and (3) ongoing psychoactive treatment. Patients were included as in- or out-patients in four sites between November 2010 and December 2015: the rehabilitation departments or outreach teams in Saint-Maurice Hospitals (Saint-Maurice), Clemenceau (Strasbourg), Capucins (Angers), or SMAEC (Lyon), France. All participants were French and had French as their mother tongue.

### Material

#### Demographic, injury severity, and intellectual functioning measures

Age at injury, time since injury and information related to injury severity were retrieved from the medical files. Several measures of injury severity were used: Glasgow Coma Scale (GCS) score (Teasdale and Jennett, 1974), length of coma (in days), time to follow simple commands (in days), and time to demutization (in days). TBI severity was defined as *moderate* if the initial/lowest GSC score was 9–12 (e.g., no coma) and/or brain lesions were seen on acute imaging; and *severe* if the initial/lowest GSC score was 3–8, and/or neurological condition leading to intubation, neuro-sedation, and mechanical ventilation in the intensive care unit. We used the number of years of education of both parents as a proxy for SES. Intellectual ability was assessed using the age-appropriate Wechsler Intelligence Scales (WISC IV; Wechsler, 2005). In this paper, we used only two subtests, reflecting verbal and visual–spatial abilities: *Vocabulary* and *Matrix Reasoning* (*Mean = 10; Standard Deviation = 3*).

#### Executive functioning measures

**Figure 1:**
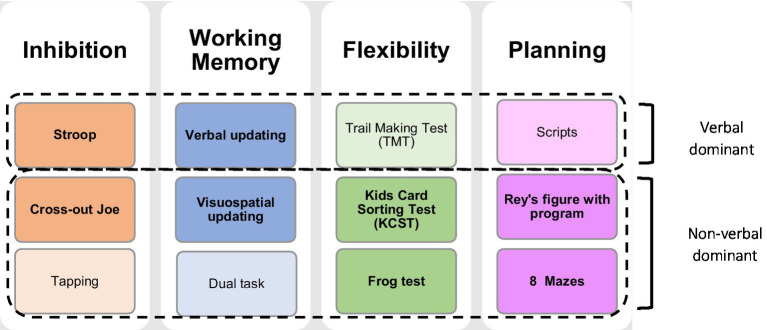
Overview of the child executive functions-battery. The main subtests per domain are highlighted in bold, and in darker colors, while complementary subtests are represented in light colors. This division was based on the factorial structure and differential analyses described in Roy et al. (2021).

Executive functions were assessed using both performance-based tests and rating scales. Regarding performance-based tests, CEF-B (Roy et al., 2021) was used to assess inhibition, working memory, flexibility, and planning abilities. CEF-B was created in France based on a child-centered theoretical developmental model of EFs (Diamond, 2013). It comprises a set of 12 performance-based tests (3 per component) designed to evaluate children and adolescents between 6 and 16 years of age (Roy et al., 2021). It comprises existing tests, modified or expanded to better target pediatric population, as well as novel experimental tasks (see Figure 1 and Table 1 for an overview and Guerra et al., 2021 for a full description of the tasks).

**Table 1:** Brief description of the tasks and measures of the CEF-B.

	Tests	Outcome variables	Description/Objective
Inhibition	Stroop	Interference time Interference errors	Consists of ignoring the reading of colored words written with non-congruent printing ink (for example, “blue” written in red), to focus on the color of the ink (interfering condition)
	Child tapping test	Go/No-Go time Go/No-Go errors Conflict time Conflict errors	Tap or not on the table depending on what the examiner is doing: (1) Go/no go: respond if the examiner taps once and inhibit if he taps twice. (2) Conflict: antagonistic conditioning (tap once if the examiner taps twice and vice versa) while incorporating a new No go condition (do not tap if the examiner taps with two fingers)
	Cross-out Joe	Time Errors	Identify and cross out a visual target (Joe) among several morphologically similar distractors on two consecutive A3 sheets
Working Memory	Verbal updating	Baseline Performance score	Sequentially recall the most recent elements (the last three or four) of a series of letters of varying length
	Visuospatial updating	Baseline Performance score	Sequentially recall the most recent items (the last three or four) touched in a series of blocks of varying length
	Dual task	Span score Clowns score Mu score	Simultaneously perform a digit span task and a visuomotor clown head crossing test
Flexibility	Child TMT	Flexibility index Alternance errors	Connect circles on a sheet of paper that contains numbers or letters, alternating numeric and alphabetical order (1-A-2-B…)
	Kids card sorting test	Time Perseverations	Initiate, maintain, and change the ranking rule of a series of 48 test cards according to four target cards that vary in three dimensions (form, color, and number), based on the examiner’s feedback
	Frog test	Time Total score	Deduce the logical rules according to which a frog moves around several water lilies placed on a pond. The child must also adapt to the actions of the frog, which changes the movement rule without previous notice
Planning	Eight mazes	Completed Total time Dead-end	The test comprises eight mazes of increasing difficulty. For each maze, a dinosaur has to find its way out. The test requires the child to draw, with a pencil, the path connecting the starting point to the maze’s exit
	Rey-Osterrieth Complex Figure (ROCF)	Planning index	Copy the ROCF spontaneously, then progressively copy the figure again, following a program consisting of five successive stages of different colors
	Scripts (Iralde et al., 2020; Allain and Iralde, 2021)	Time Sequence errors Intrusions	Put in order a sequence of phrases, elaborating a coherent script according to a given title and disconsidering those that are not relevant (intruders)

Given the large number of tasks and variables, the authors of the battery created impairment indices for each component of the battery to allow a comprehensive overview. This process was performed in four steps, namely: (1) Normalization of the data, (2) Categorization of the percentiles, (3) Calculation of the impairment index by task, and (4) Calculation of the impairment index by component (as described in Roy et al., 2021). First, and in order to consider the characteristics of the score distributions, which sometimes exhibited significant asymmetry and potentially large age effects, we used nonparametric continuous normalization (Lenhard et al., 2018). The advantage of this method is that it does not require assumptions about the parameters of the different distributions observed at each age, and it provides age-group calibration tables to calculate a percentile score directly as a function of actual age. In the second step, the percentiles obtained in the normalization process were categorized into three scores for each variable (See Table 1 for a description of the variables): (1) *Alertness score* (percentile 90–94), (2) *Impairment score* (percentile 95–98), and (3) *Severe impairment score* (percentile 99). Following this conversion, in the third step we calculated an impairment score per task, which corresponds to the average of the outcome measures scores (usually time and number of errors/success) in order to consider different aspects of each task (for a description of the results of the impairment index per task and per outcome measure in the TBI group, see Appendix 1). Finally, in the fourth step, a mean impairment index per component was created using the same calculation, which led to four composite scores overall. This calculation was performed including patients having performed at least two tasks per domain. When the child had performed all three tasks per component, we chose to consider the two main subtests of the component (see Figure 1) for comparison with the normative sample. If one of the main subtests was not administered, it was replaced by the complementary subtest. In the two last steps, the percentiles were converted to a reduced score by applying the following rule: 0 for a percentile below 80, 1 for a percentile between 80 and 89, 2 for a percentile between 90 and 94, 4 for a percentile between 95 and 98; and finally 8 for a percentile score equal to or greater than 99. Component reduced scores were the primary outcome measures. In order to obtain an individual appreciation, the reduced score was transformed into percentiles based on the normative sample.

Regarding rating scales, the parent and teacher reports of the French version of the BRIEF (Gioia et al., 2000; Roy et al., 2013) were used. The BRIEF consists of 86 items, rated using a three-point Likert scale, based on the child’s behavior occurrence: “never” (one point), “sometimes” (two points), or “often” (three points). Seventy-two of the 86 items are distributed across eight clinically and theoretically driven clinical scales measuring different aspects of EFs, and yielding’s two composite indices derived from factorial analyses: the *behavioral regulation index* (BRI: *inhibition*, *shifting*, and *emotional control*), and the *metacognitive index* (MI: *initiation*, *working memory*, *plan/organize*, *organization of materials*, and *monitor*). The *global executive composite* (GEC) *index* provides an overall measure of executive functioning. Raw scores for all scales were converted into T-scores and percentiles based on French normative data (Roy et al., 2013). The T-score was used to establish the clinical threshold in the BRIEF (T ≥ 65) while the percentiles were used to homogeneously compare results with the performances in the CEF-B.

### Procedure

The research was approved by the Savoie University ethics committee. The CEF-B and WISC-IV sub-tests were administered during a neuropsychological assessment planned for clinical or research purposes, by trained neuropsychologists using standardized instructions, in a quiet room in each child’s rehabilitation department. The BRIEF questionnaire was completed individually by a parent and/or one of the child’s teachers. All instruments were applied in French, maternal language of the children and adults.

### Statistical analyses

Scores of the TBI group on the four components of the CEF-B were compared with normative French data (Roy et al., 2021) using a *t*-test. In addition, Spearman’s rho correlations were performed to assess the degree of correlation between CEF-B components and parental/teacher ratings (BRIEF), but also among the four CEF-B components. For each patient, a dichotomous rating of congruence between CEF-B and parent/teacher ratings was conducted to determine to what extent performance-based and questionnaire-based measurements were comparable in discriminating executive deficits. For these analyses, only the scores of inhibition, working memory, shifting, plan/organize, and GEC of the BRIEF were considered (i.e., the theoretical components closest to those proposed by CEF-B). In this sense, two categories were considered: (1) Congruent scores: sum of congruent normal percentiles (<90 for both CEF-B and BRIEF) and congruent deficits (percentiles ≥90 for both CEF-B and BRIEF); (2) Incongruent deficits: BRIEF percentile ≥90 and CEF-B percentile <90 or BRIEF percentile <90 and CEF-B percentile ≥90. Based on these categories, we established an overall congruence percentage. The agreement rate was classified using Cohen’s kappa (Landis and Koch, 1977). Finally, we used Spearman’s rho correlation coefficient and *t*-test to analyze the associations between sociodemographic and injury severity variables (gender, SES, age at injury, time since injury, and intelligence tests) and executive scores. Statistical analyses were performed using SPSS v.20.0 (IBM Corp. Released, 2011). For all analyses, the significance level was set at 0.05.

## Results

### Sample and executive performance on CEF-B and BRIEF

Fifty-six patients were approached to participate in the study, four did not agree to participate and eight files were lost or too incomplete, leaving 43 patients with sufficient available data. Descriptive statistics regarding demographic and medical/TBI severity data are summarized in Table 2. Most patients had sustained severe TBI (GCS ≤8), most often following road traffic accidents or falls, with a mean length of coma of 6 days. Only three patients sustained a moderate TBI.

**Table 2:** Sample characteristics.

	**Descriptive**
N	43
Gender, *boys* (%)	28 (65.1)
Maternal education—years	
*M (SD) [Range]*	12.88 (2.9) [7–20]
Paternal education—years	
*M (SD) [Range]*	12 (4.3) [5–22]
Age at injury—years	
*M (SD) [Range]*	10.26 (3.11) [2–15]
Glasgow coma scale score	
*M (SD) [Range]*	6.07 (2.02) [3–12]
Length of coma—days	
*M (SD) [Range]*	7 (8.84) [0–43]
Time to obey simple commands—days	
*M (SD) [Range]*	9.33 (9.86) [0–43]
Time to demutization—days	
*M (SD) [Range]*	13.69 (11.69) [2–43]
Age at assessment—years	
*M (SD) [Range]*	11.45 (2.48) [7–16]
Time since injury—years	
*M (SD) [Range]*	1.23 (2.10) [0.08–9]
IQ measures	
*Vocabulary—M (SD)[Range]*	8.09 (4.48) [1–16]
*Matrix reasoning—M (SD) [Range]*	7.58 (3.80) [1–16]

SD, standard deviation; % percentages (frequencies); IQ, intellectual ability measures, from the Wechsler Intelligence Scale for Children IV.

**Figure 2:**
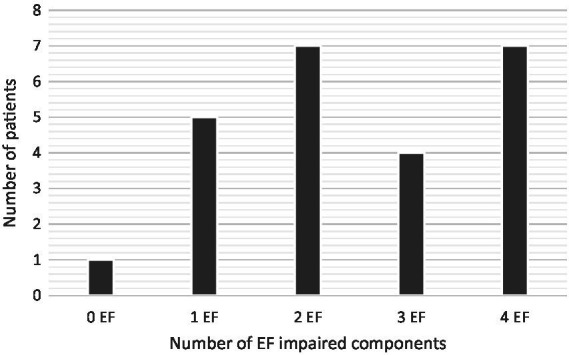
Distribution of patients according to the number of CEF-B EFs impaired components (*n* = 24).

Regarding EFs deficits, Table 3 summarizes the results of the comparison of TBI patients’ means on CEF-B components with normative data. In addition, Table 3 also presents the cumulative percentage distribution of patients with a percentile equal or greater than 90. For the whole TBI sample, the impairment indices were significantly increased in comparison with the norms, regardless of the EFs component considered. Also, findings indicate that most patients had deficits in multiple EFs domains (Figure 2) and scored beyond the 94th or the 98th percentile range compared to normative data (Table 3).

**Table 3:** Results of the CEF-B measures in the TBI group, compared to the normative data.

	TBI group	Normative data	Significance test	% Score equal or above percentile 90			
				90–94	95–98	≥99	Total
Inhibition index	2.1	0.5	*t*(33) = 5.966, *p* < 0.001				
	SD = 1.6	SD = 0.8	*d* = 1.02	14.7	14.7	32.4	61.8
	(*n* = 34)	(*n* = 935)	CI = [0.28–1.77]				
WM index	2.2	0.5	*t*(24) = 3.456, *p* < 0.001				
	SD = 2.5	SD = 0.9	*d* = 0.69	8	8	32	48
	(*n* = 25)	(*n* = 814)	CI = [−0.16–1.54]				
Flexibility index	2.6	0.4	*t*(33) = 7.043, *p* < 0.001				
	SD = 1.8	SD = 0.8	*d* = 1.21	20.6	8.8	44.1	73.5
	(*n* = 34)	(*n* = 914)	CI = [0.45–1.97]				
Planning index	2.1	0.4	*t*(30) = 5.189, *p* < 0.001				
	SD = 1.8	SD = 0.8	*d* = 0.93	16.1	19.4	29	64.5
	(*n* = 31)	(*n* = 920)	CI = [0.16–1.7]				

WM, working memory; *d*, Cohen’s effect size; and CI, confidence interval for Cohen’s *d*.

In order to evaluate correlations among CEF-B components, we performed several correlation analyses between the four impairment scores. The working memory component was significantly correlated with the inhibition component (*r* = 0.35, *p* = 0.042) and with the flexibility component (*r* = 0.52, *p* = 0.004). On the other hand, the planning component showed no significant correlations with the other components (planning and inhibition *r* = 0.27; planning and WM *r* = 0.06; and planning and flexibility *r* = 0.01). The inhibition and WM components also showed no significant correlation (*r* = 0.15).

Table 4 describes the descriptive data of the BRIEF questionnaire and the results of one-sample *t* tests based on theoretical *t*-scores. Results indicate that children with TBI had significantly elevated (T-score ≥ 65) scores in most of the clinical scales and composite BRIEF indices for teacher version (*n* = 29). In addition, the percentage of patients in the clinical range (T-score ≥ 65) was relatively high for all clinical scales, ranging from 28 to 54.5%. Regarding the parent version (*n* = 27), *t* test results were significant only for some clinical scales and indices. Furthermore, the percentage of patients in the clinical range was substantially lower than those of the teachers, ranging from 0 to 41.7%.

**Table 4:** Results of the parent and teacher ratings for the different clinical scales and composite indices of the BRIEF questionnaire and comparison to normative data.

	Parents (*n* = 27)				Teacher (*n* = 29)				Groups comparison		
	Mean (SD)	*t*	*p*	%	Mean (SD)	*t*	*p*	%	*t*	*p*	*r*
Inhibition	55.41 (14.58)	1.78	0.089	26.1	60.54 (15.57)	3.38	**0.002**	41.7	−1.35	0.192	0.286
Shifting	54.89 (13.96)	1.68	0.107	21.7	57.88 (16.71)	2.36	**0.027**	28	−1.76	0.093	0.725^**^
Emotional control	51.19 (12.08)	0.471	0.642	17.4	56.54 (15.86)	2.06	**0.050**	52	−2.37	0.028	0.704^**^
Initiation	49.63 (7.61)	0.231	0.819	4.3	64.84 (13.47)	5.51	**<0.001**	54.5	−6.14	**<0.001**	0.408
Working memory	58.71 (10.68)	3.91	**0.001**	33.3	65.70 (15.20)	5.16	**<0.001**	54.5	−1.98	0.060	0.244
Plan/organize	57.11 (12.22)	2.79	**0.011**	26.1	64.19 (16.04)	4.42	**<0.001**	50	−1.76	0.092	0.378
Organization of materials	47.61 (8.59)	1.33	0.196	0	58.94 (16.65)	2.69	**0.013**	32	−2.96	**0.007**	0.204
Monitor	55.21 (9.49)	2.63	**0.015**	14.3	61.03 (14.54)	3.79	**0.001**	44	−1.92	0.069	0.175
BRI	54.63 (13.33)	1.67	0.110	22.7	59.39 (16.26)	2.89	**0.008**	36	−2.29	0.032	0.745^**^
MI	55.27 (10.11)	2.50	**0.020**	22.7	64.77 (15.55)	4.75	**<0.001**	48	−2.73	**0.012**	0.266
GEC	55.68 (11.67)	2.33	**0.029**	41.7	64.39 (16.32)	4.41	**<0.001**	44	−2.63	**0.015**	0.400

^**^p < 0.001; BRI, Behavior regulation index; MI, Metacognition index: GEC, Global executive composite index; %, Proportion of scores in the clinical range (≥65).Values in bold are statistically significant.

### Associations between medical and sociodemographic variables and CEF-B scores

Table 5 describes exploratory *t*-test and correlation analyses performed between the scores obtained in the CEF-B components and factors known to influence EFs outcomes in the literature (age at injury, time since injury, TBI severity, e.g., here length of coma, SES, and intelligence tests). The analyses showed no significant gender differences in all four executive components and overall, most correlations were low and non-significant. Only the WISC-IV matrix reasoning subtest showed significant correlations with working memory and flexibility of the CEF-B. In addition, a significant correlation was also found between the working memory component of the CEF-B and the vocabulary subtest of the WISC-IV.

**Table 5:** Associations between results on CEF-B and demographic, injury-related and cognitive testing variables.

	CEF-B components			
	Inhibition	WM	Flexibility	Planning
Gender^a^	0.31	2.15	1.44	0.80
Level of parental education				
*Maternal education* ^b^	0.11	0.04	0.04	−0.36
*Paternal education* ^b^	0.10	0.13	−0.17	−0.39
TBI variables				
*Age at injur*y^b^	0.12	−0.11	−0.11	−0.21
*Time since injury* ^b^	0.08	−0.16	−0.23	−0.25
*Length of coma*	−0.14	−0.21	−0.14	−0.04
*Time to obey simple commands*	0.10	0.09	0.20	−0.02
*Time to demutualization*	0.15	0.06	0.11	−0.25
IQ measures				
*Vocabulary* ^b^	−0.37	−0.51^*^	−0.43	−0.41
*Matrix reasoning* ^b^	−0.34	−0.70^**^	−0.50^*^	−0.44

^a^Results referring to the *t* test (*t* value); ^b^Results regarding spearman’s rho correlations; ^*^p < 0.01. ^**^p < 0.001; TBI, Traumatic brain injury; WM, Working memory; IQ, Intellectual quotient (results of Wechsler scale subtests).

### Congruence between CEF-B components and BRIEF (parent and teacher) ratings

Correlations between parent/teacher ratings and CEF-B performances were non-significant for all clinical scales and composite scores (Table 6). However, closer examination of the rating congruence between the CEF-B and BRIEF parent and teacher forms (see Table 7) showed that 41–57% were similarly rated by parent and 50–57% by teacher as “normal” or “impaired” on the basis of the GEC. Congruence level within clinical scales was slight to moderate for teachers (39–74%; *κ* = −0.18 to 0.47), and slight to fair for parents (36–63%; *κ* = −0.16 to 0.25). When divergent, EF impairment was more frequently found in the CEF-B components than in the BRIEF parent or teacher rating.

**Table 6:** Correlation between similar CEF-B components and BRIEF clinical scales scores.

	CEF-B components			
	Inhibition	WM	Flexibility	Planning
BRIEF—Parent (*n* = 27)				
*Inhibition*	0.03	0.35	0.23	0.04
*Shifting*	−0.09	0.10	0.12	0.29
*Working memory*	−0.09	0.05	0.19	0.15
*Plan/organize*	0.08	0.02	0.12	0.26
*GEC*	−0.05	−0.03	0.14	0.24
*BRIEF*—*Teacher* (*n = 29*)				
*Inhibition*	0.01	0.34	0.27	0.24
*Shifting*	−0.08	0.43	0.22	0.21
*Working memory*	0.26	0.32	0.44	0.42
*Plan/organize*	0.18	0.26	0.17	0.28
*GEC*	−0.03	0.26	0.16	0.25

BRIEF, Behavior rating inventory of executive function questionnaire; WM, Working memory; GEC, Global executive composite score; ^*^p < 0.01, ^***^p < 0.001.

**Table 7:** Rating congruence between CEF-B components and BRIEF (parent and teacher) ratings.

CEF-B components								
	Inhibition				WM			
	*Congruent scores*	*Incongruent scores*	*Overall congruence*	*κ*	*Congruent scores*	*Incongruent scores*	*Overall congruence*	*κ*
Parent
*Inhibition*	11 (3)	10 (7)	11/21 (52%)	0.03	10 (3)	9 (6)	10/19 (52%)	0.03
*Shifting*	10 (3)	11 (7)	10/21 (48%)	0.01	12 (4)	7 (5)	12/19 (63%)	0.25
*WM*	9 (3)	12 (7)	9/21 (43%)	−0.16	11 (4)	8 (5)	11/19 (57%)	0.15
*Plan/organize*	12 (3)	9 (7)	12/21 (57%)	0.12	11 (3)	8 (6)	11/19 (57%)	0.14
*GEC*	12 (3)	9 (7)	12/21 (57%)	0.12	11 (3)	8 (6)	11/19 (57%)	0.14
*Teacher*
*Inhibition*	11 (6)	11 (6)	11/22 (50%)	0.03	11 (6)	8 (3)	11/19 (57%)	0.16
*Shifting*	10 (4)	12 (8)	10/22 (45%)	−0.18	14 (6)	5 (3)	14/19 (74%)	0.47
*WM*	11 (7)	11 (5)	11/22 (50%)	−0.03	11 (7)	8 (2)	11/19 (57%)	0.18
*Plan/organize*	14 (8)	8 (4)	14/22 (63%)	0.47	13 (7)	6 (2)	13/19 (68%)	0.37
*GEC*	11 (6)	11 (6)	11/22 (50%)	0.03	11 (6)	8 (3)	11/19 (57%)	0.16
	Flexibility				Planning			
	*Congruent scores*	*Incongruent scores*	*Overall congruence*	*κ*	*Congruent scores*	*Incongruent scores*	*Overall congruence*	*κ*
Parent
*Inhibition*	10 (6)	12 (11)	10/22 (45%)	0.08	11 (6)	12 (11)	11/23 (48%)	0.12
*Shifting*	8 (5)	14 (12)	8/22 (36%)	−0.06	11 (6)	12 (11)	11/23 (48%)	0.12
*WM*	10 (7)	12 (10)	10/22 (45%)	0.01	13 (8)	10 (9)	13/23 (56%)	0.21
*Plan/organize*	9 (5)	13 (12)	9/22 (41%)	0.05	10 (5)	13 (12)	10/23 (43%)	0.08
*GEC*	9 (5)	13 (12)	9/22 (41%)	0.05	10 (5)	13 (12)	10/23 (43%)	0.08
*Teacher*
*Inhibition*	12 (9)	11 (9)	12/23 (52%)	0.14	12 (8)	12 (9)	12/24 (50%)	0.03
*Shifting*	9 (6)	14 (12)	9/23 (39%)	−0.03	11 (6)	13 (11)	11/24 (46%)	0.05
*WM*	15 (12)	8 (6)	15/23 (65%)	0.21	15 (11)	9 (6)	15/24 (62%)	0.19
*Plan/organize*	14 (11)	9 (7)	14/23 (61%)	0.15	13 (9)	11 (8)	13/24 (54%)	0.08
*GEC*	13 (10)	10 (8)	13/23 (56%)	0.11	12 (8)	12 (9)	12/24 (50%)	0.03

κ, Cohen’s Kappa; GEC, Global executive composite. Congruent scores: sum of congruent normal percentiles (<90 for both CEF-B and BRIEF) and congruent deficits (percentiles ≥ 90 for both CEF-B and BRIEF). The number of congruent deficits is in brackets. Incongruent deficits: BRIEF percentile ≥ 90 and CEF-B percentile < 90 or BRIEF percentile < 90 and CEF-B percentile ≥ 90. The number of incongruent deficits for the CEF-B is in brackets.

## Discussion

The main objective of this study was to examine the clinical value of a new child neuropsychological performance-based tests battery (CEF-B) to explore the multidimensional nature of EFs in a moderate to severe TBI sample and to provide preliminary arguments for validity. In addition, we aimed to compare the executive performance on CEF-B to parental and teacher ratings on the BRIEF questionnaire and to investigate the influence of demographic and TBI severity variables on performance-based tests of EFs.

As expected, our findings highlight severe and global EFs deficits following severe childhood TBI, measured both by performance-based tests (CEF-B) and rating measures (BRIEF). Regarding the CEF-B, indices were significantly impaired for all executive components compared to normative data. Among the four components studied, the flexibility component was the most impaired (73.5% of the patients presented deficits), followed by planning (64.5%), inhibition (61.8%), and working memory (48%). In addition, 75% of patients were impaired in at least two EFs components and 29.2% had deficits in all four components. Impairment severity was particularly severe in this sample as most executive disorders scored in the 95 or 99th percentile range compared to normative data. Overall, our findings regarding the CEF-B provide evidence of significant executive deficits following moderate to severe TBI, as frequently reported (Babikian and Asarnow, 2009), as well as relevant data on the clinical validity of the CEF-B battery in this population.

Regarding the clinical validity of the CEF-B in childhood TBI, our findings also reveal positive and significant correlations between working memory and inhibition components and working memory and flexibility indices. Correlations between EFs factors were also found in other studies using the CEF-B in typically developing children in Brazil and in France (Guerra et al., 2021; Roy et al., 2021). These results are consistent with the literature regarding the multidimensional but interdependent character of EFs (Lehto et al., 2003; Diamond, 2013). In addition, the correlations are consistent with the propositions of Diamond (2013) who supports a progressive differentiation of EFs components throughout development. In her model, inhibition and WM are the first components to differentiate. Furthermore, the development of cognitive flexibility would also be associated with the development of the first two. Correlation between basic EFs in children post-TBI could reveal evidence that the associations between executive components follow similar principles in clinical conditions.

Although the findings of CEF-B were statistically significant and relatively homogeneous for all EF components, the rate of working memory deficits was considerably lower than the rate of deficits in the other components. These results could be related to two main issues: (1) the sample of children who underwent the working memory component sub-tests was smaller than for the other EF components (as described in Table 2, only 24 children were accounted in the analysis); (2) the working memory tasks of the CEF-B were more challenging for children with TBI than the other CEF-B tasks. Actually, the most impaired children were often not able to understand and efficiently apply the working memory tasks instructions. Therefore, the examiners decided not to propose the task systematically in order to avoid de-motivating the children. As this was not reported explicitly in the report forms, the reduced sample size for the working memory component does not allow comparing exactly the rates of impairments across EFs components, which represents a limitation of the study.

Regarding the influence of demographic and medical/TBI severity variables on CEF-B performance, correlation analyses revealed few significant associations. Actually, only some IQ measures seemed to be correlated to worse EFs. This finding confirms previous studies reporting the influence of intellectual functioning of children post-TBI on EFs measures (Vriezen and Pigott, 2003; Chevignard et al., 2010) and can also be considered as convergent with theoretical models that consider fluid intelligence as an executive component (Anderson et al., 2001; Diamond, 2013). For all other medical and sociodemographic variables, our results did not show significant correlations with CEF-B scores. Indeed, age at injury (Slomine et al., 2002; Anderson and Catroppa, 2005; Gorman et al., 2012; Chevignard et al., 2017; Krasny-Pacini et al., 2017), injury severity (Anderson and Catroppa, 2005; Conklin et al., 2008; Cooper et al., 2014; Krasny-Pacini et al., 2017; Le Fur et al., 2020) time since injury (Conklin et al., 2008), and SES (Anderson and Catroppa, 2005; Kurowski et al., 2011; Gorman et al., 2012; Ornstein et al., 2014; Krasny-Pacini et al., 2017), have been reported as factors influencing EFs outcomes in the literature, although not consistently: previous studies also reported a lack of effect of SES on EFs following severe TBI (Nadebaum et al., 2007), of age at injury (Nadebaum et al., 2007; Krasny-Pacini et al., 2017; Le Fur et al., 2020) as well as of time since injury (Chevignard et al., 2017). Family functioning and parenting style have also been reported to be associated with EF outcomes (Nadebaum et al., 2007; Kurowski et al., 2011; Anderson et al., 2012), however, unfortunately, those aspects were not assessed specifically in this study.

Concerning rating measures, we found differences between the parental and the teachers’ BRIEF ratings. Significant impairments (compared to expected norms) were found for all indices and clinical scales for the teachers’ questionnaire (11/11, with mean scores between 1 and 1.5 SD beyond the expected values, indicating severe deficits in this group as a whole), whereas only five were significant for parent ratings. Regarding parent’s reports, the GEC score was impaired in 42% of the sample, which is similar to impairment rates reported in the literature (Chevignard et al., 2012). Significant MI impairment was reported (22% impaired) but not BRI, indicating differential impairments of EF domains. The most impaired clinical scale in parent rating was Working Memory (33% impaired), similar to previous studies (see Chevignard et al., 2017). Although differences were not significant, mean scores in most of the scales and all composite indices for parent-ratings were around half a standard deviation above expected values, and the proportion of patients with scores in the clinical range was much higher than expected (mostly between 20 and 30%, as opposed to 5% in the normative sample), similar to scores reported in other prospective longitudinal samples of patients with severe TBI (Le Fur et al., 2020). Our findings confirm those of previous studies (Gioia and Isquith, 2004; Conklin et al., 2008; Chevignard et al., 2017) which reported severe working memory deficits following severe childhood TBI. However, in the current study, the overall level of impairments was lower than reported in previous studies (42–47% impaired, see Chevignard et al., 2012 for a review, and up to 65% in Chevignard et al., 2017). This could be explained by the fact that time since injury was relatively short for a large proportion of patients, who were often still undergoing intensive rehabilitation and adapted schooling following injury, as in- or out-patients. Parents had relatively recently experienced sudden trauma and life-threatening experience with the initial coma, hospitalization in the intensive care unit and medical complications. Some parents could have been more focused on motor aspects of rehabilitation, and unaware of some executive deficits evident in everyday life due to hospitalization in the rehabilitation departments (those same deficits that the specialized teachers in the hospital school reported more clearly). Also, given the recent experience of life-threatening stress, we could hypothesize that some parents were less demanding for some executive aspects of everyday life (e.g., organization), and could have attributed behavioral modifications to the hospitalization experience of their child (Roy et al., 2013).

Global and severe executive deficits were reported through teachers’ scales. Forty-four percent of the sample exhibited significant deficits on GEC score. Both MI and BRI were impaired, suggesting difficulties in all EF domains. In contrast to parents’ complaints, teachers reported severe impairment in all EF clinical scales. This is certainly also partially explained by the fact that time since injury was relatively short, in a sample of patients who had sustained mostly severe TBI, with a majority of them still hospitalized (as in- or out-patients) given their deficits, that were not compatible (yet) with rehabilitation in the community and return to school without major adaptations or even special education. Thus, specialized school was provided on-site and teachers were specialized teachers, dealing on a routine basis with children who sustained a number of conditions affecting the brain, with good knowledge and understanding of cognitive, executive, and behavioral issues following acquired brain injury. Their observation was probably accurate. This result is similar to those obtained by Chevignard et al. (2017) and suggests that dysexecutive deficits following childhood moderate to severe TBI have significant cognitive and behavioral consequences at school, which represents a demanding context in terms of attention, understanding, working memory, initiative, executive control, and behavior regulation.

Regarding associations between results of CEF-B assessment and questionnaire based BRIEF ratings, our study yielded interesting results: correlations between parent/teacher ratings and CEF-B performances were low and non-significant. This is in line with most studies having assessed correlations between direct testing and questionnaire-based reports using the BRIEF (see for example Chevignard et al., 2012; Toplak et al., 2013). This is probably due to differences in the rigorous assessment of a given skill, and the use of this skill in a changing everyday context. In this sense, it is recommended to combine direct assessment with questionnaire-based reports (if possible, in different contexts), in order to provide a better picture of the child’s functioning in various contexts (Gioia et al., 2010; Chevignard et al., 2012). On the other hand, we addressed information provided by the CEF-B and BRIEF assessments from a different angle: we measured the congruence of ratings obtained by direct assessment and questionnaire-based assessment by parents and teachers (e.g., impaired vs. non-impaired, regardless of the score itself). Using this method, it appeared that congruence between CEF-B and BRIEF scores was also slight to fair, although some indices show moderate agreement. These different types of assessments, even if not correlated, can yield reliable information in terms of presence vs. absence of deficit in the domain of executive functioning. This, in addition to the high prevalence of deficits yielded by the CEF-B, is in favor of the sensitivity of the CEF-B, despite its structured, paper and pencil format. Further, when the ratings were divergent, EF impairment was more frequently found in the CEF-B components than in the BRIEF ratings, indicating that the CEF-B tasks seem to be sensitive to executive deficits following moderate to severe childhood TBI.

This study does have a number of limitations: we used a convenience sample; patients included were all either hospitalized, either followed-up in a rehabilitation department following significant brain injury, which explains why the majority of the sample had sustained severe TBI (very few moderate TBI, who less often require such follow-up). This biased the sample toward more severe cases, unlike a prospective longitudinal study that would have included patients from the intensive care unit. Thus, our results cannot be directly translated to the whole severe TBI population. However, they contribute to the preliminary validation of the use of a newly and rigorously developed test battery to diagnose EF deficits in children who sustained moderate to severe TBI. Finally, it was not possible to measure the impact of the rehabilitation interventions that the patients received since their injury on the measures performed in this study.

In conclusion, this study reports a high prevalence of EFs deficits following childhood TBI. Although this is not a new finding, the use of the newly developed CEF-B allowed addressing deficits in a multidimensional way and addressing not only sub-test results, but also domain specific deficits, supported by factorial analyses performed in a very large normative sample. This study also shows that paper and pencil tests, addressing various EF domains, using rigorously developed standardization data, can be relatively good at exhibiting and characterizing EF deficits after severe childhood TBI. The lack of correlation with questionnaire-based measures is not a new finding and supports the recommendations to use both types of measures when assessing impairments following childhood severe TBI. The use of the CEF-B should also be explored in patients with less severe injuries and with other types of acquired or developmental brain conditions.

## Data availability statement

The raw data supporting the conclusions of this article will be made available by the authors, without undue reservation.

## Ethics statement

The study was approved by Savoie University ethics committee. The study was conducted in accordance with the local legislation and institutional requirements. Written informed consent for participation in this study was provided by the participants’ legal guardians/next of kin.

## Author contributions

MC, AR, AG, JR-R, DLG, and NF contributed to conception and design of the study. LS, EP, PN, CH, AKP, JR, and RF organized the database. AG and JL-R performed the statistical analysis. LS, MC, and AR wrote the first draft of the manuscript. MC, AR, AG, RF, and J-LR wrote the sections of the manuscript. All authors contributed to the article and approved the submitted version.
