# Supplementary material for: Comprehensive assessment of executive functioning following childhood severe traumatic brain injury: clinical utility of the child executive functions battery
Source: Front Psychol. 2023 Oct 30;14:1160210. doi: 10.3389/fpsyg.2023.1160210 (PMC10699495; doi:10.3389/fpsyg.2023.1160210)
Supplement: Supplementary file 1 [file Table_1.docx]

| **EF Component** | **Tests** | **Outcome Measure** | **N** | **Percentile ≥ 90 (%)** | **Percentile ≥ 95 (%)** | **Percentile ≥99 (%)** |  |  |
| --- | --- | --- | --- | --- | --- | --- | --- | --- |
| INHIBITION | Stroop | Interference Time | 37 | 29.7 | 21.6 | 10.8 |  | X2 = 42.219 p <.001*** |
|  |  | Interference Errors | 37 | 24.3 | 21.6 | 16.2 |  | X2 = 87.084 p <.001*** |
|  | Child Tapping Test | Go/No-Go Time | 32 | 21.9 | 15.6 | 6.3 |  | X2 = 11.733 p = 0.022* |
|  |  | Conflict Time | 32 | 43.8 | 40.6 | 34.4 |  | X2 = 361.125 p <.001*** |
|  |  | Go/No-Go Errors | 32 | 18.8 | 9.4 | 6.3 |  | X2 = 10.378 p = 0.037* |
|  |  | Conflict Errors | 32 | 25 | 21.9 | 15.6 |  | X2 = 69.875 p <.001*** |
|  | Cross-out Joe | Time | 29 | 48.3 | 24.1 | 17.2 |  | X2 = 103.069 p <.001*** |
|  |  | Errors | 29 | 41.4 | 41.4 | 31 |  | X2 = 269.142 p <.001*** |
| WORKING MEMORY | Verbal updating | Baseline | 20 | 40 | 40 | 30 |  | X2 = 173 p <.001*** |
|  |  | Performance score | 20 | 25 | 20 | 20 |  | X2 = 73.5 p <.001*** |
|  | Visuospatial updating | Baseline | 25 | 48 | 40 | 28 |  | X2 = 190.711 p <.001*** |
|  |  | Performance score | 25 | 28 | 20 | 16 |  | X2 = 57.6 p <.001*** |
|  | Dual task | Span score | 31 | 3.2 | 3.2 | 3.2 |  | X2 = 4.484 p = 0.141 |
|  |  | Clowns score | 31 | 16.1 | 9.7 | 6.5 |  | X2 = 9.52 p = 0.05. |
|  |  | Mu Score | 31 | 3.2 | 0 | 0 |  | X2 = 1.903 p = 0.6 |
| FLEXIBILITY | Child TMT | Flexibility Index | 31 | 38.7 | 35.5 | 29 |  | X2 = 247.1 p <.001*** |
|  |  | Alternance errors | 31 | 32.3 | 32.3 | 19.4 |  | X2 = 113.839 p <.001*** |
|  | Kids Card Sorting Test | Time | 27 | 59.3 | 55.6 | 51.9 |  | X2 = 705.572 p <.001*** |
|  |  | Categories | 29 | 27.6 | 3.4 | 3.4 |  | X2 = 25.138 p = 0.003** |
|  |  | Perseverations | 29 | 37.9 | 31 | 17.2 |  | X2 = 86.172 p <.001*** |
|  |  | Dropout | 29 | 17.2 | 10.3 | 0 |  | X2 = 3.586 p = 0.206 |
|  | Frog test | Time | 37 | 59.5 | 48.6 | 40.5 |  | X2 = 592.595 p <.001*** |
|  |  | Errors | 36 | 25 | 22.2 | 16.7 |  | X2 = 89.833 p <.001*** |
| PLANNING | Scripts | Time | 26 | 53.8 | 46.2 | 38.5 |  | X2 = 371.692 p <.001*** |
|  |  | Errors | 26 | 46.2 | 19.2 | 11.5 |  | X2 = 58.53 p <.001*** |
|  |  | Intruders | 26 | 11.5 | 11.5 | 3.8 |  | X2 = 4.299 p = 0.174 |
|  | ROCF | Planning Index | 33 | 24.2 | 12.1 | 12.1 |  | X2 = 46.226 p <.001*** |
|  | 8 Mazes | Total time | 35 | 40 | 28.6 | 17.1 |  | X2 = 102.429 p <.001*** |
|  |  | Dead-end | 35 | 37.1 | 28.6 | 22.9 |  | X2 = 171.222 p <.001*** |
|  |  | Completed | 40 | 47.5 | 22.5 | 20 |  | X2 = 182.875 p <.001*** |

**APPENDIX 1. Cumulative percentage of patients classified, for each component, test and outcome measure, in the alertness zone, the impairment zone or the severe impairment zone**
